# Systematic review and meta-analysis of the effects of EEG neurofeedback combined with pharmacological treatment on the positive and negative symptoms in patients with schizophrenia

**DOI:** 10.3389/fpsyt.2025.1537329

**Published:** 2025-03-28

**Authors:** Yiyi Duan, ShuFan Li, Shuqi Jia, Fen Yu, Xing Wang, Yueyu Long

**Affiliations:** Shanghai University of Sport, Shanghai, China

**Keywords:** EEG neurofeedback, pharmacological treatment, schizophrenia, positive symptoms, negative symptoms, systematic review, meta-analysis

## Abstract

**Objective:**

To evaluate the efficacy of EEG neurofeedback (EEG-NF) combined with pharmacological treatment on positive and negative symptoms in schizophrenia.

**Methods:**

Randomized controlled trials (RCTs) were searched in CNKI, Wanfang, VIP, PubMed, Web of Science, Cochrane, and Embase databases until January 25, 2025. Literature quality was assessed using the PEDro and CRED-NF checklists. Meta-analysis and publication bias tests were performed using RevMan 5.4.1 and Stata 18.0, respectively, with evidence quality evaluated via GRADEpro.

**Results:**

Fourteen studies (1371 patients) were included. EEG-NF combined with pharmacological treatment significantly improved positive (SMD=-0.87) and negative symptoms (SMD=-1.28). Subgroup analysis showed greater improvement in patients aged ≥45 years (positive: SMD=-1.05; negative: SMD=-1.64). For positive symptoms, better outcomes were observed with intervention periods ≥8 weeks, frequency ≥4 times/week, and disease duration ≥5 years (SMD=-1.04, -0.94, -0.94). For negative symptoms, better outcomes were seen with intervention periods ≥8 weeks, frequency ≥4 times/week, and disease duration <5 years (SMD=-1.34, -1.68, -1.26). Mental and emotional disorders treatment regimens targeting sensorimotor rhythm (SMR) and beta waves showed significant improvement in both positive (SMD=-0.98) and negative symptoms (SMD=-1.49).

**Conclusion:**

EEG-NF combined with pharmacological treatment effectively improves schizophrenia symptoms. A regimen of ≥4 sessions/week for ≥8 weeks, targeting SMR and beta waves, is recommended. However, publication bias may limit the generalizability of findings. Future research should prioritize larger-scale, multicenter studies to evaluate long-term efficacy and mechanisms.

**Systematic Review Registration:**

www.crd.york.ac.uk, identifier CRD42024593505.

## 1 Introduction

Schizophrenia (SCZ) is a multifaceted neuropsychiatric disorder characterized by positive symptoms (e.g., delusions, hallucinations, and hostility), negative symptoms (e.g., social withdrawal, emotional blunting), and cognitive impairments, often accompanied by enduring social dysfunction (1, 2). SCZ affects approximately 24 million individuals globally, or about 1 in every 300 people (3). According to the 2019 China Mental Health Survey, the weighted lifetime prevalence of schizophrenia and other psychotic disorders was 0.7%, placing a considerable burden on individuals, families, and society (4). Schizophrenia treatment primarily depends on the prolonged use of antipsychotic medications. Evidence suggests that antipsychotic medications are effective in alleviating positive symptoms, including agitation, aggression, and in reducing suicidal tendencies. However, their impact on improving negative symptoms remains limited (5, 6), and addressing strategies to help patients with negative symptoms reintegrate into daily life is equally important (7).

EEG neurofeedback (EEG-NF) primarily uses brainwave activity as a feedback source. In EEG-NF therapy, individual electroencephalogram signals are collected and, based on biofeedback theory, real-time feedback on psychological and physiological states is provided (8). The ability to self-regulate neural activity through neurofeedback has been shown to have potential cognitive and emotional benefits, offering greater safety, fewer side effects, and more precise modulation of relevant brain regions. This has facilitated its clinical application in a range of psychiatric disorders (9, 10). In recent years, research on neurofeedback training programs has shown benefits for various mental disorders (Depressive disorder, ADHD, Dementia, Posttraumatic stress disorder, Schizophrenia) (11–15).

Reviewing previous research, Renata Markiewicz and others used neurofeedback training to effectively improve clinical scores [Positive and Negative Syndrome Scale (PANSS)] in schizophrenic patients, with significant improvements (16). Rieger and others hypothesized that a decrease in the amplitude of the auditory-evoked N1 wave was related to AVH in schizophrenic patients, and investigated whether training to modulate the N1 component using EEG-NF would affect AVH. They found that EEG-NF training had no significant effect on N1 amplitude or AVH severity (17). N1 suppression is thought to reflect the efferent copy/corollary discharge function of the auditory system, which plays a role in the self-monitoring of speech (18, 19). Dan Cătălin Oprea et al. reviewed the preliminary results of EEG-NF as a therapeutic tool for schizophrenia in a systematic review. The main body of the research consisted mainly of case studies and case reports, which highlighted the potential use of NF as an add-on treatment option that could improve the lives of SCZ patients through changes in brain function and improvements in symptoms (20). Several studies have shown that people with schizophrenia exhibit lower alpha wave amplitudes, and there is a significant negative correlation between alpha amplitude and the severity of psychotic symptoms, especially impulsive and violent behavior. Li Zhenkuo et al. analyzed the EEG power spectrum data of schizophrenic patients with impulsive behavior, and then trained the patients in alpha wave enhancement. The results of the experimental group showed the effectiveness of neurofeedback in reducing impulsive behavior and overall psychotic symptoms (21–26). Sensorimotor rhythm (SMR) training protocols can enhance attention, with a frequency range of 12-15 Hz, which has been shown to be a beneficial frequency for anxiety (27–30). Pazooki et al. also showed that NF targeting sensorimotor rhythm (SMR) and beta-I waves could significantly improve negative symptoms and cognitive functions (31). EEG-NF therapy can effectively treat SCZ of varying degrees, improve patients’ sleep quality and anxiety (32). The results of a study by Shen et al. showed that compared with patients who only used cognitive function training, those who used EEG-NF therapy in combination with cognitive function training had higher beta and SMR waves after treatment, which helped improve the attention and learning efficiency of SCZ patients. At the same time, they also found adverse phenomena such as irritability (33). Renata Markiewicz et al. randomly divided 37 male patients with paranoid schizophrenia into a treatment group (NF, N18) and a control group (CON, N19). The treatment group received neurofeedback therapy in addition to antipsychotic drug treatment for 3 months, while the control group received standard social support. Results: After treatment, the serum concentration of reelin in the NF group was higher than that in the control group, and PANSS negative symptoms and general symptoms were significantly reduced. Neurofeedback therapy was explored from a molecular perspective as a potential mechanism in the remission process of schizophrenia (34).

A comprehensive review of the existing literature reveals a critical need for a systematic evaluation of the overall efficacy of EEG-NF in ameliorating both positive and negative symptoms in patients with schizophrenia. Furthermore, it is essential to elucidate whether a quantitative relationship exists between the therapeutic effects of EEG-NF and the improvement of these symptoms, thereby providing a robust foundation for the development of a precise intervention protocol. In light of these findings, this study employs a systematic review methodology to investigate the efficacy of EEG-NF in alleviating positive and negative symptoms in schizophrenia patients. Additionally, it seeks to determine whether the therapeutic outcomes are influenced by factors such as patient age, intervention frequency, and other relevant variables. The ultimate goal is to establish an optimized EEG-NF intervention protocol for SCZ and to generate clinically actionable evidence.

## 2 Information and methodology

### 2.1 Research framework

Based on the International Classification of Functioning, Disability, and Health (ICF) framework (35), this study examined the effects of patient age, duration of illness, and frequency and duration of EEG-NF and modes of intervention on positive and negative symptoms. The study further evaluates the effects of EEG-NF combined with pharmacotherapy on positive and negative symptoms in schizophrenia patients and investigates the dose-response relationship of EEG-NF therapy with variables such as patient age, intervention duration, and frequency. The PICO framework guiding this systematic review is presented in **Table 1**.

**Table 1:** PICO framework for EEG-NF combined with pharmacotherapy in treating positive and negative symptoms in schizophrenia patients.

Population	Intervention	Comparison	Outcome
Individuals with schizophrenia	Intervention site hospital	1. Comparison of interventions in patients of different age groups	b1positive symptoms
Age ≥18 years	intervener Doctors, nurses	2. Comparison of interventions in patients with different disease stages	b1negative symptoms
	Intervention prescription Intervention cycle	3. Comparison of intervention frequency in EEG-NF	
	Intervention duration Intervention frequency	4. Comparison of intervention periods in EEG-NF	
		5. Comparison of intervention models in EEG-NF	

### 2.2 Search strategy

Two independent reviewers performed a systematic search across seven databases, including Embase, Web of Science, PubMed, The Cochrane Library, Wanfang, VIP, and CNKI, to identify randomized controlled trials (RCTs) evaluating the combined effects of EEG neurofeedback and pharmacological treatment on the clinical symptoms of schizophrenia. The search encompassed studies published from the earliest date of database inclusion up to January 25, 2025, and additionally involved manual screening of reference lists from the included studies. The detailed literature search strategy is provided in **Table 2**.

**Table 2:** Literature search strategy.

Databases	Search procedure
Search strategy for PubMed and The Cochrane Library	#1”Neurofeedback”[Mesh] OR “alpha feedbacks” [Title/Abstract] OR “feedbacks, EEG” [Title/Abstract] OR “EEG Neurofeedback” [Title/Abstract] OR “Brainwave Biofeedback” [Title/Abstract] #2 “schizophrenia” [Mesh] OR “Schizophrenias” [Title/Abstract] OR “Dementia Praecox” [Title/Abstract] OR “Schizophrenic Disorders” [Title/Abstract] OR “Disorder, Schizophrenic “ [Title/Abstract] #3 #1 AND #2
Search strategy for Web of Science	#1 TS=(”Neurofeedback”OR”alpha feedbacks”OR”feedbacks, EEG”OR”EEG Neurofeedback”OR”Brainwave Biofeedback”) #2 TS=(”schizophrenia” OR “Schizophrenias” OR “Dementia Praecox” OR “Schizophrenic Disorders” OR “Disorder, Schizophrenic “) #3 TS=(”Randomized controlled trial” OR “Randomized” OR “Controlled” OR “Trial”) #4 #1 AND #2 AND #3
Search strategy for Embase	#1 “Neurofeedback” [exp] OR “alpha feedbacks “ [ab, ti] OR “feedbacks, EEG” [ab, ti] OR “EEG Neurofeedback” [ab, ti] OR “Brainwave Biofeedback” [ab, ti] #2 “schizophrenia” [exp] OR “Schizophrenias” [ab, ti] OR “Dementia Praecox” [ab, ti] OR “Schizophrenic Disorders”[ab, ti] OR “Disorder, Schizophrenic” [ab, ti] #3 “Randomized controlled trial” [exp] OR “Randomized” [ab, ti] OR “Controlled” [ab, ti] OR “Trial” [ab, ti] #4 #1 AND #2 AND #3
Search strategy for CNKI	Topic = (biofeedback + neurofeedback + EEG neurofeedback) AND Topic = (schizophrenia)
Search strategy for Wanfang and VIP (Vipe) databases	Topic = (biofeedback OR neurofeedback OR EEG neurofeedback) AND Topic = (schizophrenia)

### 2.3 Literature inclusion and exclusion criteria

#### 2.3.1 Inclusion criteria

(1) The study participants were clinically diagnosed with schizophrenia (≥18 years old). (2) The intervention group received electroencephalogram neurofeedback (EEG-NF) combined with pharmacological therapy. (3) The control group was treated with pharmacotherapy only. (4) The primary outcome measures included positive and negative symptoms, assessed using the Positive and Negative Syndrome Scale (PANSS), the Scale for the Assessment of Positive Symptoms (SAPS), the Scale for the Assessment of Negative Symptoms (SNAS), and the Brief Psychiatric Rating Scale (BPRS). (5) The study employed a randomized controlled trial (RCT) design.

#### 2.3.2 Exclusion criteria

(1) Repeatedly published articles. (2) The experimental group underwent combined interventions, including EEG-NF in conjunction with exercise, cognitive training, and other modalities. (3) The experimental data could not be calculated or extracted for analysis. (4) Full text could not be accessed.

### 2.4 Literature screening, data extraction and quality assessment

#### 2.4.1 Literature screening and data extraction

Upon retrieving the relevant studies, they were imported into EndNote software for duplicate removal. Subsequently, two independent researchers performed literature screening and data extraction in a double-blind manner. Data from studies meeting the inclusion criteria were entered into RevMan5.4.1 and subjected to a double-check for accuracy. In the event of disagreements, a third researcher was consulted to resolve the issue and determine whether the study should be included. The extracted information included the first author’s name, publication date, baseline characteristics of the participants (such as age, gender, and disease duration), details of the intervention, and outcome measures.

#### 2.4.2 Quality assessment

The quality of the literature was assessed using the PEDro scale (36) and the cred-nf checklist (37) to evaluate the methodological quality of the included literature, in which the PEDro scale includes “random allocation”, “allocation concealment”,”baseline similarity”, “blinding of study participants”, “blinding of therapists”, “blinding of outcome assessment” and “blinding of the therapist”. “similarity at baseline”, ‘blinding of study participants’, ‘blinding of therapists’, ‘blinding of outcome assessment’, ‘participation rate >85%’, ‘blinding of therapists’, ‘blinding of therapists’, ‘blinding of therapists’, ‘blinding of therapists’. “Participation rate >85%”, ‘Intention-to-treat analysis’, ‘Between-group statistical outcome analysis’, ‘Point-measurement measures of difference’, and 10 other items. Literature that met one of the criteria was scored as 1 point and those that did not were scored as 0. The scale was totaled to 10 points, with scores below 4 considered low quality, scores between 4 and 5 as moderate quality, 6 to 8 as higher quality, and 9 to 10 as high quality. Only literature of moderate quality and above was included.

The CRED-NF checklist provides a systematic assessment of the quality of the design and reporting of the included studies and contains Pre-experiment, Control groups, Control measures, Feedback specifications, Outcome measures, Data storage. This scale delivers a holistic overview of the methodological rigor and reporting quality of the included studies.

In addition, the quality of evidence for each outcome indicator was assessed using the GRADEpro system. The quality of evidence for each outcome indicator was categorized into four levels: high, medium, low, and very low. Quality ratings were completed independently by two researchers, and in the event of disagreement, a third researcher would intervene to discuss the matter until agreement was reached.

### 2.5 Data processing

Heterogeneity of all outcome measures from the included studies was analyzed using RevMan5.4.1 software, incorporating sample size, as well as the mean and standard deviation of pre- and post-intervention improvement values. As all included outcome measures were continuous variables, mean difference (MD) was used for analysis when measurement methods and units were consistent, whereas standardized mean difference (SMD) was employed when measurement methods or units differed. Heterogeneity was evaluated using P-values and I². Significant heterogeneity was considered when the P-value was less than 0.05 and I² exceeded 50%, in which case a random effects model was applied. Conversely, if the P-value was greater than or equal to 0.05 and I² was less than or equal to 50%, no significant heterogeneity was observed, and a fixed effects model was used. The results of the meta-analysis are reported with 95% confidence intervals (95% CI). Publication bias was assessed using Stata 18.0 software.

## 3 Results

### 3.1 Literature search results

**Figure 1:**
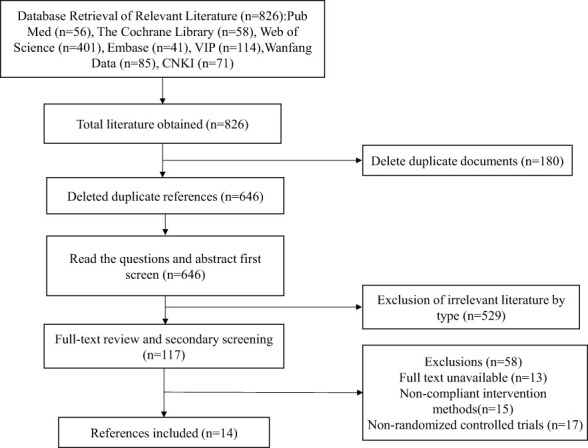
Literature screening process.

A total of 826 relevant studies were identified, with 180 duplicates removed. Following title and abstract review, 529 studies were excluded, leaving 117 studies for further assessment. In accordance with the inclusion and exclusion criteria, 14 studies were finally included, as illustrated in **Figure 1**.

### 3.2 Basic information on the studies included

This study included 14 studies, comprising 1,371 participants, with 685 in the experimental group and 686 in the control group. All participants were diagnosed with schizophrenia, and the included studies were published between 2015 and 2024. All included studies employed EEG neurofeedback combined with medication in the experimental group, while the control group received medication alone. These studies provided comprehensive details on the intervention protocols, including frequency, duration, and session length. Specifically, the intervention duration varied from 4 to 12 weeks, with a frequency of 2 to 5 sessions per week, and each session lasting 20 to 30 minutes. The basic characteristics of the included studies are summarized in **Table 3**.

**Table 3:** Basic information of included studies.

Included Studies	Age (years)		Course of an illness (years)		EEG-NF protocol details	Intervention duration, frequency, cycle	Feedback modality	Disease severity	Evaluation tool
	T/C (Sample size)		T/C (Sample size)						
Zhou Fangzhen 2015 (38)	31.83 ± 9.64(n=53)	32.62 ± 9.03(n=52)	6.46 ± 2.18(n=53)	8.05 ± 3.07(n=52)	Mode of operation: Mood and emotional disorders mode C3 in the central area and the prefrontal area β (13 to 32 HZ) combined with SMR wave (12 to 15 HZ) training	20~30min/t 2t/w 8w	Visual	Patients with chronic schizophrenia, duration of illness > 2 years; Brief Psychiatric Rating Scale (BPRS) score ≥ 36 points	①
GuoYanting 2022 (39)	35.3 ± 5.5(n=70)	35.6 ± 5.6(n=69)	6.51 ± 1.24(n=70)	6.45 ± 1.22(n=69)	Mode of operation: Mood and emotional disorders mode β、θ、SMR wave	20~30min/t 4t/w 8w	Visual and auditory	Schizophrenia; duration of illness 2-11 years	②
Lin Caiting 2023 (40)	57.32 ± 2.46(n=43)	57.65 ± 2.24(n=43)	4.43 ± 1.56(n=43)	4.53 ± 1.24(n=43)	Mode of operation: Mood and emotional disorders mode C3 C4	20~30min/t 4t/w 8w	⎯⎯	Chronic schizophrenia, duration of illness 1-10 years	②
Qiyan 2019 (41)	37.72 ± 6.30(n=80)	36.81 ± 7.76(n=80)	7.24 ± 1.36(n=80)	7.15 ± 1.62(n=80)	Mode of operation: Mood and emotional disorders mode Prefrontal C3 β and SMR wave	20min/t 5t/w 4w	Visual and auditory	Schizophrenia Course of illness 1–14 years	①
Chen Xiao 2017 (42)	39.93 ± 9.42(n=30)	39.93 ± 10.7(n=30)	5.43 ± 2.55 month(n=30)	5.30 ± 2.45 month(n=30)	Prefrontal area Central area	30min/t 3t/w 8 w	⎯⎯	Schizophrenia with predominant negative symptoms PANSS score > 60 points, negative symptom scale ≥ 60 points	②
JuXuan 2021 (43)	62.12 ± 8.46(n=41)	53.26 ± 1.34(n=41)	7.41 ± 1.60(n=41)	4.76 ± 1.45(n=41)	Mode of operation: Mood and emotional disorders mode Central C3 β and SMR wave	20~30min/t 2t/w 8 w	⎯⎯	Patients with chronic schizophrenia complicated by tardive dyskinesia have a chronic schizophrenia course of ≥2 years and have been taking antipsychotic drugs for ≥3 months; at least 2 items on the abnormal involuntary movement rating scale (AIMS) score ≥2 or 1 item scores ≥3.	①
Li JIE 2023 (44)	39.87 ± 6.90(n=45)	40.11 ± 6.81(n=45)	⎯⎯	⎯⎯	Participant specific protocol	20min/t 3t/w 12 w	Visual and auditory	First-episode schizophrenia, first onset, and none have received antipsychotic drugs or electroconvulsive therapy; total score on the positive and negative syndrome scale (PANSS) >60 points, and risk assessment for running away, impulsivity, and running away is moderate risk and above	②
Wang Hongyan 2020 (45)	35.72 ± 4.83(n=49)	37.63 ± 5.26(n=51)	4.05 ± 2.11(n=49)	3.89 ± 1.35(n=51)	Participant specific protocol	20min/t 5t/w 4 w	⎯⎯	Schizophrenia, duration of illness ≥ 2 years	②
Jing Wenming 2022 (46)	45.00 ± 1.28(n=60)	45.00 ± 1.20(n=60)	6.00 ± 0.66(n=60)	6.00 ± 0.57(n=60)	Mode of operation: Mood and emotional disorders mode C3 β and SMR wave	20min/t 5t/w 4 w	Visual and auditory	Chronic schizophrenia, duration of illness 1–11 years	③
Sun Zhiyong 2015 (47)	28.5 ± 7.1(n=32)	27.5 ± 6.7(n=33)	1.2 ± 0.5(n=32)	1.1 ± 0.6(n=33)	Participant specific protocol	20min/t 3t/w 6 w	Visual and auditory	Schizophrenia, duration ≤ 2 years, no prior use of antipsychotic medication; PANSS score > 60 in all cases	②
Wang Tianming 2022 (48)	35.5 ± 7.2(n=40)	35.3 ± 7.0(n=40)	⎯⎯	⎯⎯	Prefrontal area Participant specific protocol	⎯⎯ ⎯⎯ 8 w	⎯⎯	First-episode schizophrenia	②
Ren Hong 2020 (49)	37.28 ± 1.42(n=52)	37.31 ± 1.38(n=52)	7.31 ± 0.48 month(n=52)	7.29 ± 0.52 month(n=52)	Vp1, Vp2, Vpz, alpha wave, theta wave, SMR wave the main parameters are: electrode impedance < 10 k ohms, current < 10 μV, voltage 16-20Hz	30min/t 5t/w 8 w	⎯⎯	Schizophrenia BPRS score ≥ 36 points	③
LiuBangwen 2023 (50)	32.76 ± 6.37(n=40)	31.52 ± 8.95(n=40)	4.98 ± 2.01(n=40)	5.25 ± 1.23(n=40)	β wave, θ wave, SMR wave Participant specific protocol	20min/t 4t/w 6 w	⎯⎯	Schizophrenia. Those with no history of antipsychotic drugs or other forms of related treatment within 4 weeks before treatment	①
Chenhaibing 2024 (52)	32.92 ± 4.59(n=50)	32.89 ± 4.57(n=50)	12.18 ± 1.36month(n=50)	12.14 ± 1.34 month(n=50)	Keep θ waves in control 4~8 Hz, β wave control at 15~20 Hz	30 min/t ⎯⎯ 8w	⎯⎯	Schizophrenia: Positive and Negative Symptom Scale (PANSS) score ≥60 points	②

T, Experimental group; C, Control group; ‘⎯⎯’, not reported; w, week; t, time.

①BPRS(Brief Psychiatric Rating Scale**)**②PANSS(Positive and Negative Syndrome Scale)③SANS(Scale for the Assessment of Negative Symptoms)and SAPS (Scale for the Assessment of Positive Symptoms).

### 3.3 Literature quality assessment

All 14 studies included in this analysis met the following criteria: “baseline similarity,” “participant rate > 85%,” “intention-to-treat analysis,” “intergroup statistical analysis,” and “point measurements and difference values.” Nine studies were conducted using “random allocation” and received PEDro scores ranging from 5 to 7, with a mean score of 5.8. No studies of low quality were identified, indicating that the overall quality of the included studies was high, as shown in **Table 4**.

**Table 4:** Literature quality assessment.

Included Studies	A1	A2	A3	A4	A5	A6	A7	A8	A9	A10	Totals
ZhouFangzhen 2015 (38)	1	0	1	0	0	0	1	1	1	1	7
Guo Yanting 2022 (39)	1	0	1	0	0	0	1	1	1	1	6
Lin Caiting 2023 (40)	1	0	1	0	0	0	1	1	1	1	6
Qi Yan 2019 (41)	1	0	1	0	0	0	1	1	1	1	6
Liu Bangwen 2023 (50)	0	0	1	0	0	0	1	1	1	1	5
Chen Xiao 2017 (42)	1	0	1	0	0	0	1	1	1	1	6
Ju Xuan 2021 (43)	1	0	1	0	0	0	1	1	1	1	6
Li Jie 2023 (44)	0	0	1	0	0	0	1	1	1	1	5
WangHongyan 2020 (45)	0	0	1	0	0	0	1	1	1	1	5
Jing Wenming 2022 (46)	1	0	1	0	0	0	1	1	1	1	6
Ren Hong 2020 (49)	0	0	1	0	0	0	1	1	1	1	5
Sun Zhiyong 2015 (47)	1	0	1	0	0	0	1	1	1	1	6
Wang Tianming 2022 (48)	1	0	1	0	0	0	1	1	1	1	6
Chen Haibing 2024 (52)	1	0	1	0	0	0	1	1	1	1	6

A1, random allocation; A2, Assignment hiding; A3, Baseline similarity; A4, Blindness of the study population; A5, Therapist blindness; A6, Results-based assessment of blindness; A7, Participation rate > 85 percent; A8, Intention-to-treat analysis; A9, Analysis of statistical results between groups; A10, Point measurements and difference values.

This study systematically assessed the quality of experimental design and reporting in neurofeedback research using the CRED-nf checklist. The checklist outlines best practices for the design and reporting of neurofeedback studies, aimed at advancing the understanding of brain mechanisms related to neurofeedback. The highest scores in the CRED-nf checklist were obtained in the categories of “Control Groups and Control Measures”, “Feedback Specifications”, and “Outcome Measures”. Furthermore, the low scores in the “Pre-experiment” and “Data Storage” categories indicate a persistent lack of transparent research practices, such as study registration and data sharing. Detailed scores for each study are provided in **Table 5**.

**Table 5:** Consensus on the Reporting and Experimental Design of clinical and cognitive behavioral Neurofeedback studies (CRED-nf) best practices checklist 2020.

		Ren Hong 2020 (49)	Liu Bangwen 2023 (50)	Zhou FangZhen 2015 (38)	Sun ZhiYong 2015 (47)	Jing WenMing 2022 (46)	Li Jie 2023 (44)	Lin Caiting 2023 (40)	Wang Tianming 2022 (48)	Wang Hongyan 2020 (45)	Ju Xuan 2021 (43)	Guo Yanting 2022 (39)	Chen Xiao 2017 (43)	Qi Yan 2023 (41)	Chen Haibing 2024 (52)	Percent meeting best practice
	Domain															
**1**	1.1	**-**	**-**	**-**	**-**	**-**	**-**	**-**	**-**	**-**	**-**	**-**	**-**	**-**	**-**	**0％**
	1.2	**-**	**-**	**＋**	**＋**	**＋**	**-**	**＋**	**＋**	**-**	**＋**	**＋**	**-**	**＋**	**＋**	**64.3％**
**2**	2.1	**＋**	**＋**	**＋**	**＋**	**＋**	**＋**	**＋**	**＋**	**＋**	**＋**	**＋**	**＋**	**＋**	**＋**	**100％**
	2.2	**-**	**-**	**-**	**-**	**-**	**-**	**-**	**-**	**-**	**-**	**-**	**-**	**-**	**-**	**0％**
	2.3	**-**	**-**	**-**	**-**	**-**	**-**	**-**	**＋**	**-**	**-**	**-**	**-**	**-**	**-**	**7.1％**
	2.4	**-**	**-**	**-**	**-**	**-**	**-**	**-**	**-**	**-**	**-**	**-**	**-**	**-**	**-**	**0％**
	2.5	**＋**	**＋**	**＋**	**＋**	**＋**	**＋**	**＋**	**＋**	**＋**	**＋**	**＋**	**＋**	**＋**	**＋**	**100％**
**3**	3.1	**-**	**-**	**-**	**-**	**-**	**-**	**-**	**-**	**-**	**-**	**-**	**-**	**-**	**-**	**0％**
	3.2	**＋**	**＋**	**＋**	**＋**	**＋**	**＋**	**＋**	**＋**	**＋**	**＋**	**＋**	**＋**	**＋**	**＋**	**100％**
	3.3	**-**	**-**	**-**	**-**	**-**	**-**	**-**	**-**	**-**	**-**	**-**	**-**	**-**	**-**	**0**
	3.4	**-**	**-**	**-**	**-**	**-**	**-**	**-**	**-**	**-**	**-**	**-**	**-**	**-**	**-**	**0**
	3.5	**-**	**-**	**-**	**-**	**-**	**-**	**-**	**-**	**-**	**-**	**-**	**-**	**-**	**-**	**0**
**4**	4.1	**＋**	**＋**	**＋**	**-**	**＋**	**-**	**＋**	**-**	**-**	**＋**	**＋**	**-**	**＋**	**＋**	**64.3％**
	4.2	**＋**	**-**	**-**	**-**	**-**	**＋**	**-**	**-**	**-**	**＋**	**＋**	**-**	**＋**	**＋**	**42.9％**
	4.3	**-**	**-**	**-**	**＋**	**＋**	**＋**	**-**	**-**	**-**	**-**	**＋**	**-**	**＋**	**-**	**35.7％**
	4.4	**-**	**-**	**-**	**-**	**-**	**-**	**-**	**-**	**-**	**＋**	**-**	**-**	**-**	**-**	**7.1％**
	4.5	**＋**	**＋**	**＋**	**＋**	**＋**	**＋**	**＋**	**＋**	**＋**	**＋**	**＋**	**＋**	**＋**	**＋**	**100％**
**5**	5.1	**＋**	**＋**	**＋**	**-**	**-**	**-**	**＋**	**-**	**-**	**-**	**＋**	**-**	**-**	**＋**	**35.7％**
	5.2	**-**	**-**	**-**	**-**	**-**	**-**	**-**	**-**	**-**	**-**	**-**	**-**	**-**	**-**	**0％**
	5.3	**＋**	**＋**	**＋**	**＋**	**＋**	**＋**	**＋**	**＋**	**＋**	**＋**	**＋**	**＋**	**＋**	**＋**	**100％**
	5.4	**＋**	**-**	**-**	**-**	**＋**	**＋**	**-**	**＋**	**-**	**-**	**＋**	**＋**	**-**	**＋**	**50％**
	5.5	**-**	**-**	**-**	**-**	**-**	**-**	**-**	**-**	**-**	**-**	**-**	**-**	**-**	**-**	**0％**
**6**	6.1	**-**	**-**	**-**	**-**	**-**	**-**	**-**	**-**	**-**	**-**	**-**	**-**	**-**	**-**	**0％**
	**Percent criteria met by study**	**39.1％**	**30.4％**	**34.8％**	**30.4％**	**39.1％**	**34.7％**	**34.7％**	**34.7％**	**21.7％**	**39.1％**	**47.8％**	**26.1％**	**39.1％**	**43.5％**	**35.2％**

1Pre-experiment 1.1Pre-register experimental protocol and planned analysis1.2Justify sample size 2Control groups 2.1Employ control group(s) or condition(s) 2.2When leveraging experimental designs where a double-blind is possible, use a double blind 2.3Blind those who rate the outcomes 2.4Examine to what extent participants and experimenters remain blind 2.5In clinical efficacy studies, employ a standard of care intervention group as a benchmark for improvement 3Control measures 3.1Collect data on psychosocial factors 3.2Report whether participants were provided with a strategy 3.3Report the strategies participants used 3.4Report methods used for online-data processing and artifact correction 3.5Report condition and group effects for artifacts 4Feedback specifications 4.1Report how the online-feature extraction was defined 4.2Report and justify the reinforcement schedule 4.3Report the feedback modality and content 4.4Collect and report all brain activity variable(s) and/or contrasts used for feedback, as displayed to experimental participants 4.5Report the hardware and software used 5Outcome measures 5.1Report neurofeedback regulation success based on the feedback signal 5.2Plot within-session and between session regulation blocks of feedback variable(s), as well as pre-to-post resting baselines or contrasts 5.3Statistically compare the experimental condition/group to the control condition(s)/group(s) (not only each group to baseline measures) 5.4Include measures of clinical or behavioral significance, defined *a priori*, and describe whether they were reached 5.5Run correlational analyses between regulation success and behavioral outcomes 6Data storage 6.1Upload all materials, analysis scripts, code, and raw data used for analyses, as well as final values, to open access data depository.

Percent meeting best practice column at right-percent of those studies meeting the particular domain best practice; Percent criteria met by study row at bottom - percent of all domains being met by the particular study.

### 3.4 Meta-analysis results

#### 3.4.1 Fourteen studies investigated positive symptoms, with the results of the random-effects model meta-analysis

**Figure 2:**
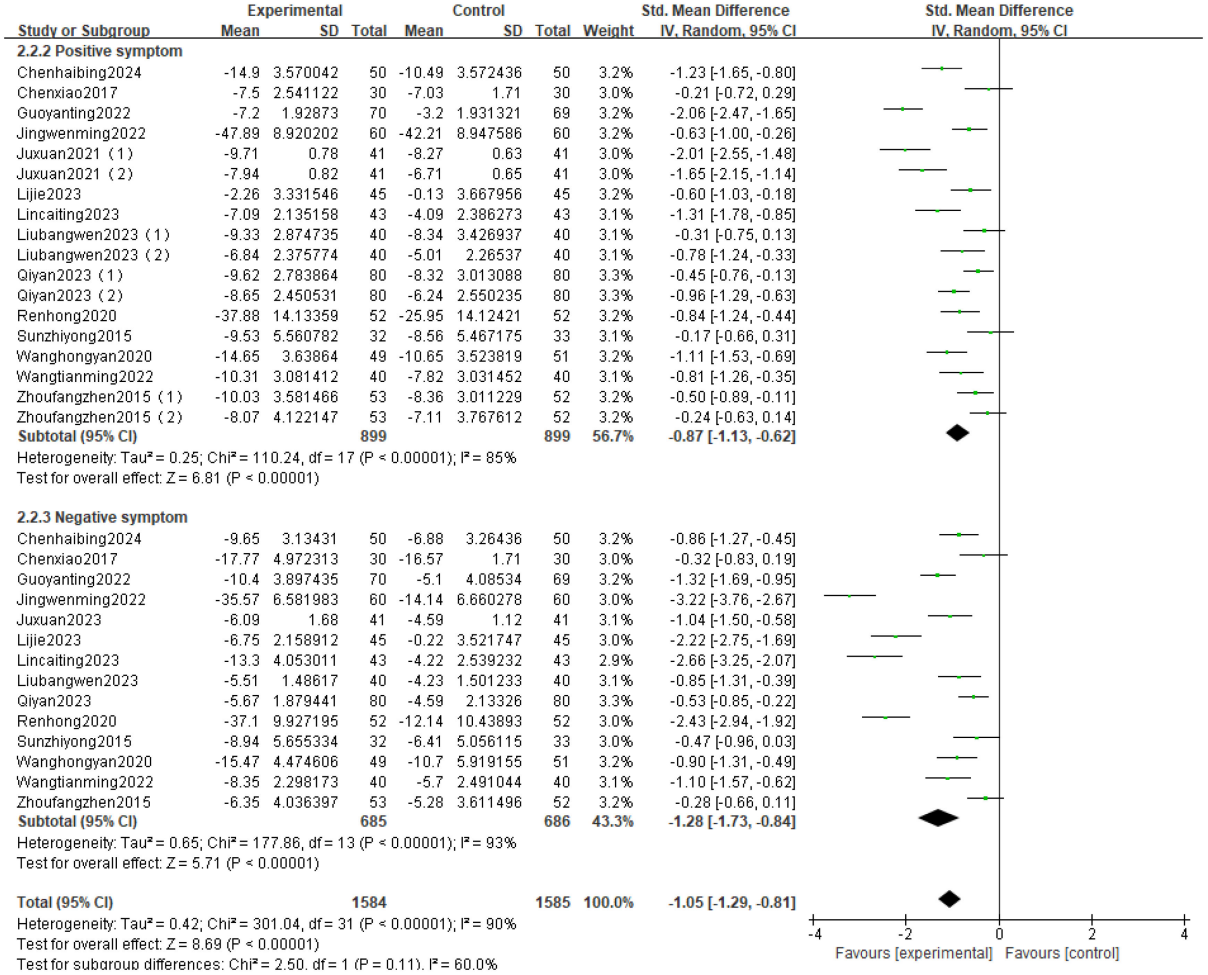
Random-effects meta-analysis forest plot for positive and negative symptoms.

The intervention group showed significantly greater improvement in positive symptoms in schizophrenia patients compared to the control group, with a statistically significant difference [SMD = -0.87, 95% CI (-1.13, -0.62), P < 0.001] (**Figure 2**).

#### 3.4.2 Fourteen studies investigated negative symptoms, with the results of the random-effects model meta-analysis

The intervention group showed a significantly greater improvement in negative symptoms in schizophrenia patients relative to the control group, with a statistically significant difference [SMD = -1.28, 95% CI (-1.73, -0.84), P < 0.001] (**Figure 2**).

#### 3.4.3 Subgroup analysis

To investigate potential sources of heterogeneity, subgroup analyses of positive and negative symptoms were performed, as shown in **Table 6**. The effectiveness of EEG neurofeedback combined with medication on positive and negative symptoms in patients with schizophrenia (SCZ) may be influenced by factors such as the mode of intervention and the period of intervention. The studies were divided into two subgroups based on the mean age of patients: ≥45 years and <45 years. According to the mean course of disease, the studies were divided into two subgroups: ≥5 years and <5 years. the intervention frequency was classified as ≥4 times/week and <4 times/week. The intervention duration was divided into two subgroups: ≥8 weeks and <8 weeks. The intervention models can be grouped into mental and emotional disorders model and other models(participant-specific agreements). The results of the subgroup analysis in **Table 6** indicated that both the positive and negative symptom measures were statistically significant.

**Table 6:** Results of Meta-analysis of the effects of EEG neurofeedback combined with pharmacological treatment on positive and negative symptoms in patients with schizophrenia.

Outcome indicator		Number of studies included	I²/%	Meta-analysis results	
				SMD (95%CI)	P-value
Positive symptom		14(1798)	85	-0.87(-1.13,-0.62)	<0.001
Age (Mean age)	≥45years <45years	6(589) 8(1209)	84 86	-1.05(-1.48,-0.61) -0.77(-1.08,-0.46)	<0.001 <0.001
Disease duration(Mean value)	≥5years <5 years	6(1113) 6(471)	90 80	-0.94(-1.34,-0.55) -0.74(-1.17,-0.31)	<0.001 0.0008
Frequency of intervention	≥4 t/w <4 t/w	7(1029) 5(589)	85 88	-0.94(-1.27-0.60) -0.76 (-1.26,-0.26)	<0.001 0.003
Intervention period	≥8 w <8 w	9(1033) 5(765)	88 62	-1.04(-1.42,-0.65) -0.64(-0.88,-0.40)	<0.001 <0.001
Intervention model	Mood and mental disorders pattern Other	6(986) 8(759)	86 63	-0.98(-1.34,-0.62) -0.69(-0.93,-0.44)	<0.001 <0.001
Negative symptom		14(1371)	93	-1.28(-1.73,-0.84)	<0.001
age (Mean age)	≥45 years <45 years	6(507) 8(864)	91 93	-1.64(-2.35,-0.94) -1.02(-1.56,-0.49)	<0.001 <0.001
Duration disease(Mean value)	≥5 years <5 years	6(686) 6(515)	94 93	-1.19(-1.89,-0.49) -1.26(-2.00,-0.53)	0.0009 0.0008
Frequency of intervention	≥4 t/w <4 t/w	7(789) 5(402)	95 90	-1.68(-2.41,-0.96) -0.86(-1.53,-0.18)	<0.001 0.01
Intervention period	≥8w <8 w	9(846) 5(525)	92 95	-1.34(-1.88,-0.81) -1.18(-2.02,-0.33)	<0.001 0.006
Intervention model	Mood and mental disorders pattern Other	6(692) 8(679)	96 89	-1.49(-2.32,-0.66) -1.14(-1.63,-0.64)	0.0004 <0.001

#### 3.4.4 Sensitivity analysis

**Figure 3:**
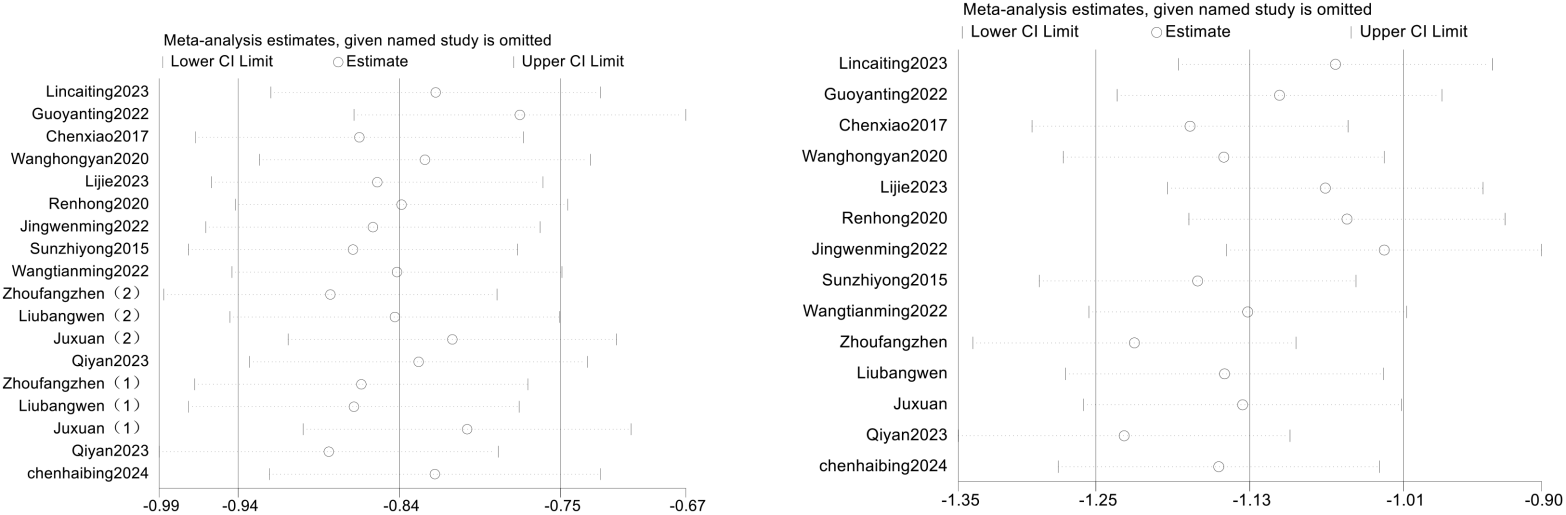
Bubble chart for sensitivity analysis of positive and negative symptoms.

To explore whether heterogeneity between studies was driven by any single study, a sensitivity analysis was performed on the effects of EEG-NF combined with pharmacotherapy on positive and negative symptoms in schizophrenia patients. The combined effects were analyzed by sequentially excluding individual studies, as shown in **Table 7** and **Figure 3**. After excluding the study by Guo Yanting et al. (39), the combined effect for positive symptoms was SMD = -0.80, 95% CI (-1.02, -0.58), P < 0.001. The I² decreased from 85% to 79%, indicating reduced heterogeneity, and the difference remained statistically significant when compared to the control group. After excluding other individual studies, the combined effect SMD ranged from -0.94 to -0.76, and the I² ranged from 81% to 86%, with all P values < 0.001. Sequential exclusion of individual studies did not result in a significant reduction in heterogeneity for negative symptoms. The combined effect SMD ranged from -1.36 to -0.97, with I² ranging from 90% to 93%, and all P values < 0.001.

**Table 7:** Combined effect of excluding individual study-positive and negative symptoms.

	Exclusion study	Effect size	95%CI	P(merger effect)	I²/%
	Ren Hong 2020 (49)	-0.88	-1.14, -0.61	<0.001	85
	Liu Bangwen 2023 (50)	-0.92	-1.19, -0.64	<0.001	86
	Zhou Fangzhen 2015 (38)	-0.94	-1.21, -0.67	<0.001	84
	Sun Zhiyong 2015 (47)	-0.91	-1.17, -0.66	<0.001	84
	Jing wenming 2022 (46)	-0.89	-1.16, -0.62	<0.001	85
Positive symptom	Li Jie 2023 (44)	-0.89	-1.16, -0.63	<0.001	85
	Lin Caiting 2023 (40)	-0.85	-1.11, -0.59	<0.001	85
	Wang Tianming 2022 (48)	-0.88	-1.14, -0.61	<0.001	85
	Wang Hongyan 2020 (45)	-0.86	-1.13, -0.60	<0.001	85
	Ju Xuan 2021 (43)	-0.76	-1.00, -0.53	<0.001	81
	Guo Yanting 2022 (39)	-0.80	-1.02, -0.58	<0.001	79
	Chen Xiao 2017 (42)	-0.91	-1.17, -0.65	<0.001	85
	Qi Yan 2023 (41)	-0.90	-1.19, -0.61	<0.001	85
	Chenhaibing 2024 (52)	-0.85	-1.12, -0.59	<0.001	85
	Ren Hong 2020 (49)	-1.20	-1.63, -0.76	<0.001	92
	Liu Bangwen 2023 (50)	-1.32	-1.79, -0.85	<0.001	93
	Zhou Fangzhen 2015 (38)	-1.36	-1.82, -0.91	<0.001	92
	Sun Zhiyong 2015 (47)	-1.35	-1.81, -0.88	<0.001	93
	Jing Wenming 2022 (46)	-0.97	-1.52, -0.75	<0.001	90
	Li Jie 2023 (44)	-1.21	-1.66, -0.77	<0.001	93
	Lin Caiting 2023 (40)	-1.18	-1.61, -0.75	<0.001	92
Negative symptoms	Wang Tianming 2022 (48)	-1.30	-1.77, -0.83	<0.001	93
	Ju Xuan 2023 (43)	-1.30	-1.78, -0.83	<0.001	93
	Wang Hongyan 2020 (45)	-1.32	-1.79, -0.84	<0.001	93
	Guo Yanting 2022 (39)	-1.28	-1.77, -0.80	<0.001	93
	Chen Xiao 2017 (42)	-1.36	-1.82, -0.90	<0.001	93
	Qi Yan2023 (41)	-1.35	-1.82, -0.87	<0.001	93
	Chenhaibing2024 (52)	-1.32	-1.80,- 0.84	<0.001	93

### 3.5 Publication bias

**Figure 4:**
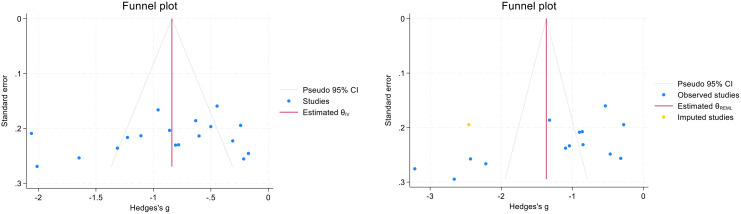
Positive and negative symptom indicators publication bias.

In this study, Stata18.0 was used to analyse the positive and negative symptom outcome indicators for publication bias, and Egger’s test for the positive symptom indicators: P> |t| = 0.243>0.05 suggests that there is no obvious publication bias; for the negative symptom indicators: P> |t| = 0.011<0.05, and the non-parametric cut-and-patch method of analysis for publication bias was used to find that there was no significant change in the pre- and post-effects as well as the confidence intervals, suggesting an obvious publication bias. confidence intervals were not significantly changed, suggesting obvious publication bias, see **Figure 4**.

### 3.6 Evaluation of the quality of evidence

The GRADEPro software indicates that the quality of evidence for both positive and negative symptoms is rated as moderate and low, as presented in **Table 8**. This result may have limitations related to the lack of allocation concealment or blinding in some studies as well as publication bias for baseline inconsistency and negative symptoms.

**Table 8:** GRADE evaluation of the quality of evidence.

Outcome indicator	Included Studies	Evaluation of the quality of evidence					Quality of evidence
		Research Limitations	Inconsistency	Indirectness	Inaccuracy	Publication bias	
Positive symptom	14	severity	not serious	not serious	not serious	not serious	middle level
Negative symptom	14	severity	not serious	not serious	not serious	severity	low level

## 4 Discussion

The results of this study indicate that EEG-NF therapy, in combination with medication, improved both positive and negative symptoms in schizophrenia patients, consistent with findings from previous studies. Rieger et al. demonstrated that neurofeedback training significantly enhances single-trial auditory evoked potentials (AEPs) in individuals with schizophrenia. The enhancement of AEPs was correlated with a reduction in auditory verbal hallucinations (AVH), suggesting that NF may mitigate symptoms by modulating auditory neural processing. These findings support the potential of NF as a viable therapeutic strategy for individuals with schizophrenia (17). EEG neurofeedback therapy applies directional stimulation to the nervous system via simulated EEG currents, strengthens positive feedback mechanisms, promotes β-wave activity, and optimizes the distribution of the brain wave power spectrum. It also facilitates the repair of damaged brain cells by improving brain circulation and regulates parasympathetic nervous system function to enhance neuromodulation (51). Second-generation antipsychotics, including risperidone, which binds to 5-HT and DA receptors in the brain to alleviate positive symptoms (53), may also cause adverse effects. With prolonged use, patients may continue to experience symptoms such as slowed emotional expression, poor concentration, and an increased risk of diabetes mellitus (52, 54). The combination of EEG-NF therapy with psychotropic medication enhances therapeutic efficacy, offering greater effectiveness and safety compared to medication alone (55).

This study also found that EEG neurofeedback combined with medication was more effective in improving positive and negative symptoms in SCZ patients aged ≥45 years. Older patients tend to exhibit more pronounced negative symptoms, possibly due to a longer duration of schizophrenia and extended hospitalization, both of which are associated with the development of “hospitalization syndrome,” exacerbating the severity of negative symptoms (56). For SCZ patients with a disease duration of ≥5 years, EEG-NF therapy may be more effective in improving positive symptoms. In contrast, for SCZ patients with a disease duration of <5 years, EEG-NF therapy may be more effective in improving negative symptoms. In addition, this study found that EEG-NF therapy, when administered for ≥8 weeks and with a frequency of ≥4 sessions per week, had a more significant effect on improving both positive and negative symptoms. Previous studies have demonstrated that improvements in brain function during EEG-NF training are associated with changes in brainwave patterns within specific regions, and that the frequency of specific brainwaves increases with the number of interventions, exhibiting a dose-dependent relationship (57, 58). Intensive EEG neurofeedback training, conducted over a short period, can successfully modulate feedback characteristics and yield improvements in target behaviors (59, 60). Therefore, increasing the treatment volume by extending the number of intervention cycles and the frequency of sessions within each cycle was more effective in improving both positive and negative symptoms in SCZ patients. Negative symptoms are more pronounced in older patients, with greater sensitivity to intervention, leading to more pronounced improvements. Additionally, portable EEG devices have been shown to yield results comparable to state-of-the-art wired EEG systems in event-related paradigms, supporting mobile use. A home-based EEG neurofeedback study for chronic pain treatment demonstrated that active EEG-NF had at least a moderate clinical effect (≥30%) on improving mean pain scores on the Brief Pain Scale (61). These findings may be of interest to patients with schizophrenia and clinicians and may inform the design of future EEG-NF trials.

This paper also conducted a subgroup analysis of EEG-NF training modalities, which were categorized into two groups: one group used the mood and mental disorders model, with SMR and βwaves training, and the other group used other training modalities (participant-specific protocols). The results showed that the effect of electroencephalographic neurofeedback targeting sensorimotor rhythms (SMR) and βwaves on the improvement of positive and negative symptoms of schizophrenia was significantly higher than in the group with the Control group, a finding that is consistent with negative symptoms of the study of Pazooki et al. (31), who reported that SMR/β-waves training improves thalamo-cortical loop functional connectivity through modulation of thalamo-cortical loop functional connectivity (fMRI evidence of FC enhancement of the MFG to the thalamus) emotional apathy and social withdrawal, supporting the pathomechanism specificity of targeted neurofeedback.

This study included a total of 14 studies, systematically evaluating and analyzing the effects of EEG neurofeedback combined with pharmacological treatment on both positive and negative symptoms in patients with schizophrenia. The quality of the included studies was assessed using the PEDro scale, yielding an average score of 5.8. No low-quality studies were identified, indicating an overall high quality of the literature. Key factors influencing the quality scores included allocation concealment, blinding of participants, blinding of therapists, and blinding of outcome assessors. Meta-analysis revealed that the I² values for both positive and negative symptoms exceeded 50%, indicating a high degree of heterogeneity. Consequently, we assessed the included studies and identified significant variations in EEG-NF protocols, including differences in target brain regions, specific brain waves trained, and associated frequencies, which likely contributed to the observed heterogeneity. Among the 14 included studies, 57.1% reported target frequency bands, while only 14.3% provided transparent details on target frequencies [e.g., Chen et al. trained theta waves at 4-8 Hz and beta waves at 15-20 Hz (52); Zhou et al. trained beta waves at 13-20 Hz and SMR waves at 12-15 Hz (38)]. To enhance the comparability of results, we recommend that future studies explicitly report target brain regions, frequency bands, and frequencies. Furthermore, variations in baseline characteristics across studies may partially account for the observed heterogeneity. Among these, six studies provided detailed baseline severity data, and seven reported additional information, such as the type and duration of diagnosed schizophrenia. Four studies, for instance, reported a history of psychiatric medication use. The lack of comprehensive baseline data may have obscured important sources of efficacy heterogeneity, making the current conclusions more applicable to mixed populations with unknown baseline characteristics. For positive symptoms, sensitivity analyses failed to identify specific sources of heterogeneity. However, subgroup analyses revealed that categorizing studies by intervention duration (≥8 weeks vs. <8 weeks) and intervention mode (mode of mental and emotional disorders vs. other modes) significantly reduced heterogeneity, with I² values of 88% and 62% for duration, and 86% and 63% for mode, respectively. These results were statistically significant, suggesting a potential dose-response relationship between intervention duration/mode and symptom improvement. The residual heterogeneity may be attributed to variations in EEG-NF protocol design and the baseline severity of patients’ illness. Subgroup and sensitivity analyses for negative symptoms did not identify specific sources of heterogeneity.

In this study, the quality of evidence rating was performed using GRADEpro software, and the effectiveness of EEG neurofeedback combined with medication in intervening on positive and negative symptoms in patients with schizophrenia was given intermediate and low quality of evidence. Study limitations and publication bias were downgrading factors: most of the literature did not fully report on blinding or did not implement allocation concealment, which may have introduced some limitations to the studies. A publication bias test was performed for positive and negative symptoms indicators, and the results showed that there may be publication bias for negative symptoms. The possible reason for publication bias is that all studies did not meet the first requirement of the CRED-NF checklist for pre-registration. The Registered Report (RR) is an innovative publication format distinguished by its focus on peer review of the study protocol rather than the study results (62, 63). If the protocol is approved during peer review, the study is guaranteed publication irrespective of the significance of its results. This format promotes the publication of non-significant findings, thereby enhancing the stability and reliability of scientific research. Therefore, Registered Reports can be used to reduce the risk of selective reporting bias by “prioritizing methodological review”. For example, when Trambaiolli et al. applied the CRED-NF checklist to evaluate NF interventions in the field of cognitive aging, they found that some of the studies still lacked study registration and data sharing, emphasizing the importance of transparent research practices (14). Also, Voigt et al. in their comprehensive review in the field of psychiatry highlighted the need for standardized feedback protocols and data transparency (13). It is therefore recommended that future NF studies adopt pre-registration and RR as standard practice. In addition, we encourage researchers to use the EEG protocol template to improve methodological consistency and transparency (64).

This study also had the following limitations: 1. The limited number of included literature and the fact that most studies did not fully report the details of the EEG-NF protocol and information such as baseline disease severity, etc., and major symptoms, end could stratify these influences for further study, limiting the ability to determine the most effective method. 2. The difficulty of making the EEG neurofeedback intervention process double-blind resulted in the actual amount of combined effect of the clinical significance needs to be interpreted with caution. 3. Negative symptoms are particularly susceptible to publication bias, potentially leading to an overestimation of the effect size even after correction using the trim-and-fill method, thereby limiting the generalizability of the findings. Given the limited availability of relevant literature, this study did not impose pre-registration as an inclusion criterion for selected studies, which may have contributed to publication bias. In order to improve the robustness and generalisability of the results of future studies, it is strongly recommended to provide registered reports for intervention studies.

## 5 Conclusion

EEG neurofeedback combined with pharmacological treatment improves both positive and negative symptoms in schizophrenia patients, and there is a dose effect of improvement with patient age, disease duration, and the mode, period, and frequency of EEG neurofeedback. An intervention program of EEG neurofeedback in schizophrenia patients with mental and mood disorder intervention modalities (targeting SMR waves and β-waves) ≥4 times per week for ≥8 weeks is recommended. It is important to highlight that there is a publication bias in the intervention effect of EEG neurofeedback combined with pharmacological treatment on negative symptoms in patients with schizophrenia, potentially limiting the generalizability and reliability of the findings. Future research should prioritize larger-scale, multicenter studies to comprehensively evaluate the long-term efficacy and underlying mechanisms of EEG neurofeedback combined with medication in the treatment of schizophrenia.

## Data Availability

The original contributions presented in the study are included in the article/supplementary material. Further inquiries can be directed to the corresponding author.
